# Molecular analyses of two bacterial sampling methods in ligature‐induced periodontitis in rats

**DOI:** 10.1002/cre2.98

**Published:** 2018-02-15

**Authors:** Carla Raquel Fontana, Clovis Grecco, Vanderlei Salvador Bagnato, Laura Marise de Freitas, Constantinos I. Boussios, Nikolaos S. Soukos

**Affiliations:** ^1^ Universidade Estadual Paulista (Unesp), Faculdade de Ciências Farmacêuticas Araraquara SP Brazil; ^2^ Instituto de Física‐Grupo de Óptica–Universidade de Sao Paulo, USP SP Brazil; ^3^ Laboratory for Information and Decision Systems Massachusetts Institute of Technology, MIT Massachusetts USA; ^4^ Department of Physics 111 Dana Research Center Massachusetts USA

**Keywords:** biofilm, periodontal pathogens, molecular diagnostics, sampling technique

## Abstract

The prevalence profile of periodontal pathogens in dental plaque can vary as a function of the detection method; however, the sampling technique may also play a role in determining dental plaque microbial profiles. We sought to determine the bacterial composition comparing two sampling methods, one well stablished and a new one proposed here. In this study, a ligature‐induced periodontitis model was used in 30 rats. Twenty‐seven days later, ligatures were removed and microbiological samples were obtained directly from the ligatures as well as from the periodontal pockets using absorbent paper points. Microbial analysis was performed using DNA probes to a panel of 40 periodontal species in the checkerboard assay. The bacterial composition patterns were similar for both sampling methods. However, detection levels for all species were markedly higher for ligatures compared with paper points. Ligature samples provided more bacterial counts than paper points, suggesting that the technique for induction of periodontitis could also be applied for sampling in rats. Our findings may be helpful in designing studies of induced periodontal disease‐associated microbiota.

## 1 INTRODUCTION

Dental plaque is a complex microbial biofilm in which more than 700 species of bacteria have been identified (Dewhirst et al., 2010). Pathogenic microorganisms were found in subgingival plaque samples obtained from periodontally healthy and diseased subjects (Haffajee et al., 1998). Studies of periodontal disease‐associated microbiota usually analyze bacterial plaque to describe the contents of the periodontal pocket using different microbial sampling techniques (Casas et al., 2007; Guentsch et al., 2011; Persson, Weibel, Hirschi, & Katsoulis, 2008). However, there are extensive variations in the application of sampling methods and considering the importance of subgingival plaque bacteria in the etiology, diagnosis, and treatment of periodontitis, reliable sampling methods are needed. In humans, sampling of subgingival bacteria is more common with curettes or paper points (Persson et al., 2008; Teles, Haffajee, & Socransky, 2008). Sampling of dental plaque has also been reported with cotton swab (Barsamian‐Wunsch, Park, Watson, Tinanoff, & Minah, 2004; Beikler et al., 2006). Baker, Butler, and Wikesjö (1991), Fine (2009), Graves, Fine, Teng, Van Dyke, and Hajishengallis (2008), Klausen (1991), Tanner and Goodson (1986) discussed sampling using curettes, scalers, paper points, broaches within cannula, and irrigation of periodontal pockets. They stated that paper points were used by an increasing number of investigators mostly for microbiological culture studies; hereby, the loosely adherent tissue associated microorganisms in the periodontal pocket were sampled.

Numerous experimental models in animals such as rats, hamsters, canines, ferrets, rabbits, and primates have been developed in order to reproduce periodontal diseases (gingivitis and periodontitis; Fine, 2009; Graves et al., 2008; Struillou, Boutigny, Soueidan, & Layrolle, 2010). In rats, experimental periodontitis may be obtained by using silk ligatures tied around the molars or by inoculations of specific microbial species (Guessous et al., 1994; Klausen, 1991). The destructive phase of ligature‐induced experimental periodontitis is the result of the interaction of bacterial plaque and host immune response locally—as in human periodontitis—that leads to the formation of an inflammatory infiltrate in the adjacent gingival tissue and the subsequent destruction of connective tissue and bone (Trindade et al., 2014). Although experimental periodontitis induced in rats is the most commonly used model, to the best of our knowledge, no studies have been published on the microbial profile of ligature‐induced periodontitis in rats, in recent decades. Besides, no studies have compared the efficacy of different sampling techniques for the assessment of the subgingival microbiota in rats. Due to the extensive variation of sampling methods, the investigation of ligatures potential as a sampling technique is noteworthy: If ligatures themselves could be employed as the sampling method, experimental periodontitis in rats would be shortened in one step, reducing variability.

In the present study, our goal was to use a ligature‐induced periodontitis model in rats in order to compare the subgingival microbiota in plaque samples obtained directly from ligatures or from periodontal pockets using absorbent paper points. Our hypothesis was that ligature samples would provide a similar plaque composition and a greater number of bacterial counts, thus suggesting the superiority of this sampling technique over that of using paper points. If the hypothesis were true, this would mean that the ligature could be used as both a method of periodontitis induction and sample collection, thus eliminating one step of the sampling procedure and preventing variation.

## 2 MATERIALS AND METHODS

### 2.1 Animals

Thirty male Wistar rats with mean weight of 200 g were used. Four rats were housed in each cage and maintained under 12‐hr light/dark cycle at a temperature of 25 ± 2 °C and relative humidity of 50% with access to standard rat chow pellets and water ad libitum. Before all operative procedures, the animals were anesthetized by an intramuscular injection of Ketamine hydrochloride (Ketamin 10 ml; Virbac do Brasil Ind. Com. Ltda, Roseira, SP, Brazil) at a dose of 0.10 ml/100 g body weight, associated with a muscular relaxant and analgesic Coopazine (Xylazine, Schering‐Plough Saude Animal Ind Com. Ltda, Cotia, SP, Brazil) at a dose of 0.05 ml/ 100 g body weight. The study protocol (# 0509) was reviewed and approved by the Ethics and Research Committee at the Medical School of Ribeirao Preto–University of Sao Paulo.

### 2.2 Experimental induction of periodontitis

Following anesthesia, sterile 2/0 cotton ligatures (*Polycot* ®, Johnson e Johnson, São Paulo, SP, Brazil) were placed around the cervix of the right first inferior molar. The rats were immobilized at a custom made surgical table, allowing a reasonable mouth opening to place the cotton cord. The first right inferior molar was assigned to receive a ligature in a cervical position. The thread was introduced in the proximal space between the first and second molar with two small hemostatic clamps to hold the cotton cord. Two knots were made on the mesial face of the first molar and the ligatures were kept in position in order to allow plaque accumulation and pocket formation over a period of 27 days, according to Semenoff, Semenoff, Borges, Pedro, and Sakai (2010).

Periodontitis induction was confirmed by histology. Briefly, the tooth and surrounding soft tissue were removed in block and fixed in 10% formalin, decalcified for 45 days in Morse solution prepared by mixing equal volumes of 20% sodium of citrate (*w*/*v*) and 50% formic acid (*v*/v). Following dehydration (alcohol 70%—1 hr, 90%—1 hr, and 100%—16 hr) and paraffin embedding (Histoembedder ® Leica), 6 μm thick longitudinal sections in the mesiodistal direction were obtained and stained with hematoxylin and eosin (H/E) technique. The sections were examined histologically under a light microscope.

### 2.3 Bacterial sampling

Twenty‐seven days after the ligature placement, rats were anesthetized and microbiological samples were obtained directly from the ligatures as well as from the periodontal pockets using absorbent sterile paper points. In each animal, one sample was obtained from the ligature and another one from the periodontal pocket. All samples were obtained by the same dentist in order to standardize the sampling procedure. First, ligatures were carefully removed with scissors and dental tweezer and then transferred immediately into a transport sterile microtube. In a sequence, and in the same tooth, four sterile paper point ISO (International Organization for Standardization) #40 (DiaDent Group INternational Burnaby, BC, Canada) were inserted as deeply as possible into the pocket, one at each tooth site (mesial, lingual, distal, and facial), for 20 s and then all four paper points were transferred immediately into the same microtube. At the end of experimental procedures, all ligatures and paper point samples were individually placed in microtubes containing 0.15 ml TE (10 mM Tris–HCl, 1 mM EDTA, pH 7.6). Subsequently, 0.1 ml of freshly prepared NaOH (0.5 M) was added to each tube and the samples were homogenized using a vortex mixer. Microbial analysis was carried out in the Applied Molecular Photomedicine Laboratory at The Forsyth Institute, Boston using whole genomic probe analysis as described below (Socransky et al., 1994).

### 2.4 Microbial analysis

Samples were lysed, and the DNA was placed in lanes on a positively charged nylon membrane using a Minislot device (Immunetics, Cambridge, MA, USA). After fixation of the DNA to the membrane, the membrane was placed in a Miniblotter 45 (Immunetics) with the lanes of DNA perpendicular to the lanes of the device. Digoxigenin‐labeled whole genomic DNA probes to 40 bacterial species were hybridized in individual lanes of the Miniblotter. After hybridization, the membranes were washed at high stringency and the DNA probes were detected using an antibody against digoxigenin conjugated with alkaline phosphatase for chemifluorescence detection. Signals were detected using AttoPhos substrate (Amersham Life Science, Arlington Heights, IL, USA) and were scanned with a Storm Fluorimager (Molecular Dynamics, Sunnyvale, CA, USA), a computer linked instrument that read the intensity of the fluorescent signals resulting from the probe–target hybridization. Two lanes in each run contained standards at concentrations of 10^5^ and 10^6^ cells of each species. The sensitivity of the assay was adjusted to permit detection of 10^4^ cells of a given species by adjusting the concentration of each DNA probe. Signals were evaluated using the Storm Fluorimager and were converted to absolute counts by comparison with the standards on the same membrane. Failure to detect a signal was recorded as zero.

### 2.5 Statistical analysis

Data from samples were not parametric (*p* < .05 for Shapiro–Wilk normality test); therefore, the significance of the difference between ligature and sulcus sample was determined with the Mann–Whitney test using GraphPad Prism® Version 5.01 software (GraphPad Software Inc., La Jolla, CA, USA). Differences were considered to be significant when *p* < .05 (confidence level of 95%).

## 3 RESULTS

### 3.1 Histology

**Figure 1:**
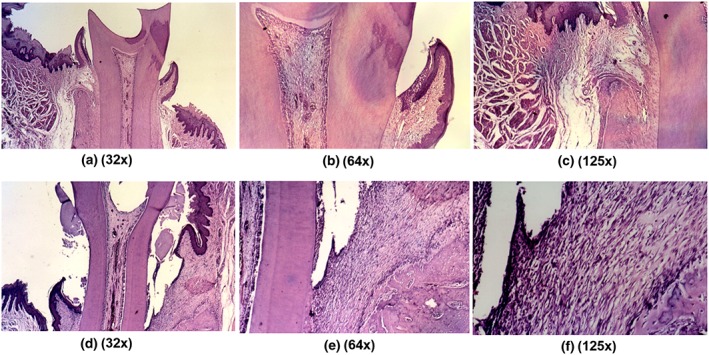
Histological analyses of longitudinal sections in the mesiodistal direction**. (a–c)** General view of the normal relationship between tooth structures and periodontal tissues. The following observations were made: absence of periodontal pockets with disruption of the junctional epithelium; presence of intact epithelium; absence of inflammation in the subcutaneous tissue; absence of bone resorption; and presence of normal distribution of the periodontal fibers. H/E, 32×, 64×, and 125×, respectively. (**d–f)** After induction of periodontitis, periodontal pockets, epithelium disruption, intense inflammatory infiltrate, and severe bone resorption were observed. H/E, 32×, 64×, and 125×, respectively

**Figure 2:**
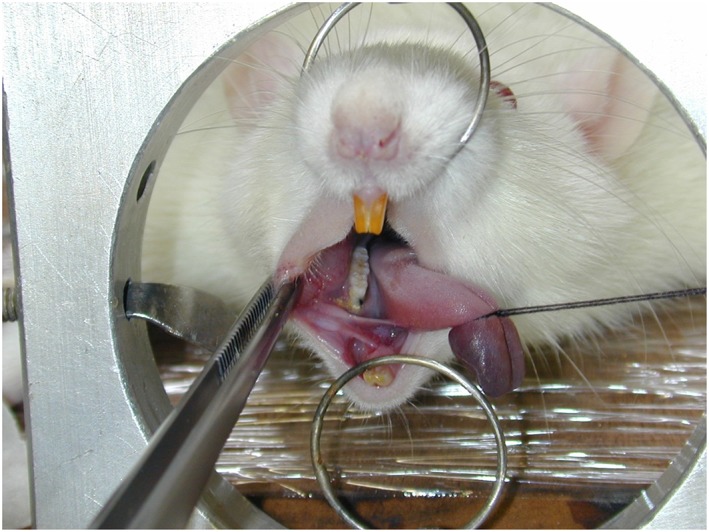
A custom made surgical table used to attach the ligature to the first molar

Results from H&E staining demonstrates the controls (Figure 1a–c), with normal connective and histological characteristics with obvious no inflammatory infiltration. Following experimental induction of periodontitis, the inflammatory reaction was intense with severe bone resorption (Figure 1d–f). Figure 2 shows the ligature in place and the surgical table used in this protocol.

### 3.2 Detection frequency of periodontal bacteria

**Figure 3:**
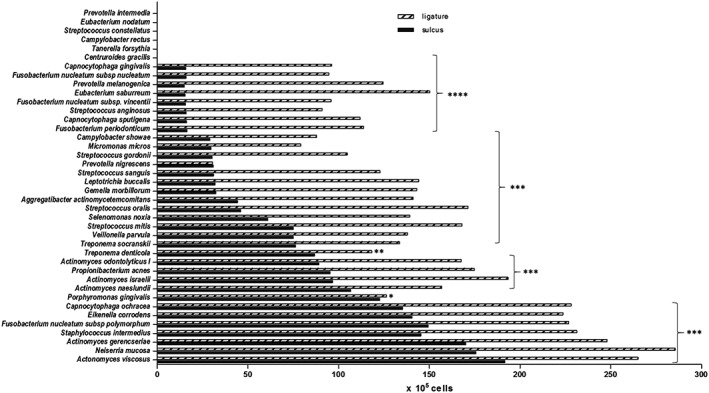
Bacterial detection levels in ligature (black bar) and paper point (pattern bar) samples. The horizontal axis shows DNA probe counts of 40 oral bacteria (10^5^ cells). The vertical axis shows bacterial species. Higher amounts of bacteria were collected from ligatures than with paper points. Statistical analysis revealed a significant difference between sulcus and ligature sampling for all species. Mann–Whitney test; *p < .05; **p < .01; ***p < .001; ****p < .0001

Table 1 presents the frequency of bacterial detection for both sampling techniques. Figure 3 shows levels (DNA probe counts) of 40 bacteria in dental plaque samples obtained from ligature as well as those obtained with paper points.

**Table 1:** Frequency of bacterial detection in ligature samples and paper point samples

Bacterial species (periodontal complex color)	Frequency of detection	
	Ligature sample	Sulcus sample
*Prevotella intermedia* (O)	**0**	**0**
*Eubacterium nodatum* (O)	**0**	**0**
*Streptococcus constellatus* (O)	**0**	**0**
*Campylobacter rectus* (O)	**0**	**0**
*Tanerella forsythia* (R)	**0**	**0**
*Centruroides gracilis* (O)	**0**	**0**
*Capnocytophaga gingivalis* (G)	**2**	**2**
*Fusobacterium nucleatum subsp nucleatum* (O)	**5**	**2**
*Prevotella melanogenica* (O)	**6**	**1**
*Eubacterium saburreum*	**6**	**1**
*Fusobacterium nucleatum subsp. vincentii* (O)	**6**	**1**
Streptococcus anginosus	**6**	**1**
*Capnocytophaga sputigena* (G)	**6**	**2**
*Fusobacterium periodonticum* (O)	**7**	**1**
*Campylobacter showae* (O)	**7**	**1**
*Micromonas micros* (O)	**7**	**2**
*Streptococcus gordonii* (Y)	**8**	**1**
*Prevotella nigrescens* (O)	**8**	**2**
*Streptococcus sanguis* (Y)	**8**	**5**
*Leptotrichia buccalis*	**8**	**5**
*Gemella morbillorum*	**8**	**8**
*Aggregatibacter actinomycetemcomitans* (G)	**8**	**6**
*Streptococcus oralis* (Y)	**9**	**1**
*Selenomonas noxia* (Y)	**9**	**2**
*Streptococcus mitis* (Y)	**9**	**2**
*Veillonella parvula* (P)	**9**	**3**
*Treponema socranskii*	**9**	**4**
*Treponema denticola* (R)	**10**	**7**
*Actinomyces odontolyticus I* (P)	**11**	**3**
*Propionibacterium acnes*	**11**	**5**
*Actinomyces israelii* (B)	**11**	**6**
*Actinomyces naeslundii* (B)	**11**	**6**
*Porphyromonas gingivalis* (R)	**12**	**6**
*Capnocytophaga ochracea* (G)	**13**	**9**
*Eikenella corrodens* (G)	**14**	**9**
*Fusobacterium nucleatum subsp polymorphum* (O)	**14**	**10**
*Staphylococcus intermedius* (Y)	**15**	**10**
*Actinomyces gerencseriae* (B)	**15**	**11**
*Neiserria mucosae*	**16**	**11**
*Actonomyces viscosus* (B)	**17**	**13**

*Note*. B = blue complex; G = green complex; O = orange complex; P = purple complex; R = red complex (Socransky, Haffajee, Cugini, Smith & Kent 1998). Unpaired *t*‐test, *p* < .0001.

The bacterial composition patterns were similar for both sampling methods with the detection levels in periodontal pocket samples being markedly lower compared with those in ligature samples. More specifically, the same 34 species were detected in both ligature and periodontal pocket samples (Table 1), but ligature samples included significantly higher amounts of bacteria than periodontal pocket samples (Figure 3), the threshold of detection being 10^5^ cells.

In ligature samples, the detection frequency of 34 microorganisms ranged from 6.6% to 56.6%, whereas in periodontal pocket samples the detection frequency ranged from 3.3% to 43.3%. Six microorganisms (*Campylobacter rectus*, *Streptococcus constellatus*, *Eubacterium nodatum*, *Campylobacter gracilis*, *Tannerella forsythia*, and *Prevotella intermedia*) were not detected at all; and only *Porphyromonas gingivalis* and *Prevotella nigrescens* showed similar detection frequencies in both ligature and paper point samples (Figure 2).

Our results show that the dominant species were early colonizers. These included *Actinomyces viscosus*, *Actinomyces gerencseriae*, *Streptococcus intermedius*, *Streptococcus mitis*, *Streptococcus oralis*, *Capnocytophaga ochracea*, and *Eikenella corrodens*.

## 4 DISCUSSION

The aim of the present study was to use a ligature‐induced periodontitis model in rats in order to investigate the prevalence profile of periodontal pathogens in dental plaque samples obtained directly from ligatures or from periodontal pockets using absorbent paper points. The ligature‐induced periodontitis model was used because it mimics features of human periodontitis, including the formation of an inflammatory infiltrate, loss of attachment, and loss of alveolar bone. The majority of studies have kept the ligature in place from 15 to 60 days for the induction of periodontal destruction (Çalışır, Akpınar, Poyraz, Göze, & Çınar, 2015; de Molon et al., 2015; Fontana, Kurachi, Mendonça, & Bagnato, 2004; Johnson, 1975). In this study, ligatures were removed 27 days after their placement. Microbial analysis was performed using DNA probes to a panel of 40 periodontal species in the checkerboard assay, an efficient technique for detection of periodontal bacteria in supragingival and subgingival plaque samples (Socransky et al., 1994). Our data obtained from the analysis of the sample plaque composition showed that the same 34 periodontopathogens were recovered with both ligature and paper points with early colonizers being the dominant species. Six bacteria were not detected at all. Detection levels for all species were markedly lower for paper points than for ligatures with the exception of *Porphyromonas gingivalis* and *Prevotella nigrescens* that showed similar detection frequencies in both ligature and paper point samples.

The prevalence profile of periodontal pathogens in dental plaque can vary as a function of the detection method; however, the sampling technique may also play a role in determining dental plaque microbial profiles. Paper points are widely established for the collection of subgingival plaque or other samples to analyze oral microbiota. ISO 45 paper points were proven to work most efficiently, whereas sampling times between 5 and 30 s did not reduce the sampling efficiency (Hartroth et al., 1999). In our study, we used ISO 40 paper points and the sampling time was 20 s. In the present study, the bacterial composition patterns were similar for both ligature and paper point sampling. This clearly suggests that the ligature‐induced periodontitis model could also be applied for sampling in rats. The finding that ligature samples provided more bacterial counts than paper points may undergo different interpretations. It is possible that the sampling sequence did not exhibit any effect. Paper points were used after the removal of ligatures to avoid disturbance of biofilms developed on ligatures, and ligature collection did not influence the succeeding one with paper points. In this case, literature may offer explanations why paper points collected fewer bacteria. Specifically, it has been reported that paper points are used for sampling loosely adherent tissue associated microorganisms in the periodontal pocket (Tanner & Goodson, 1986). Loomer (2004) reported that paper points collect plaque from the outer layer of the plaque. At the same time, paper points are less successful at sampling the apical part of the pocket, where more pathogens are expected to be. This result was partially confirmed by Baker et al. (1991) in their in vitro study testing whether paper points sampled homogenous and nonhomogenous plaque equally from all parts of periodontal pockets. They concluded that paper points misrepresented the composition of microbial communities in the apical part of periodontal pockets. It is also possible that biofilms on ligatures remained intact following their removal, but ligature collection disturbed biofilms in periodontal pockets. As a consequence, paper points collected fewer bacteria. The weakness of the present study is that it cannot demonstrate the effect of the sampling sequence. The latter should be investigated in future studies with the appropriate design.

In conclusion, the results of the present study have demonstrated that even though ligature samples harvested significantly more bacteria, the composition of the plaque samples with respect to selected target pathogens were quite similar for both sampling techniques. This is the first study to demonstrate that ligature could be used as both a method of periodontitis induction and sample collection. Our findings may be helpful in designing studies of induced periodontal disease‐associated microbiota.

## CONFLICT OF INTEREST

The authors declare that there are no conflicts of interests.

## ETHICAL APPROVAL

The study protocol # 0509 was reviewed and approved by the Ethics and Research Committee at the Medical School of Ribeirao Preto–University of Sao Paulo.

## References

[cre298-bib-0001] Baker, P. J. , Butler, R. , & Wikesjö, U. (1991). Bacterial sampling by absorbent paper points. An in vitro study. Journal of Periodontology, 62(2), 142–146. 2027062 10.1902/jop.1991.62.2.142

[cre298-bib-0002] Barsamian‐Wunsch, P. , Park, J. H. , Watson, M. R. , Tinanoff, N. , & Minah, G. E. (2004). Microbiological screening for cariogenic bacteria in children 9 to 36 months of age. Pediatric Dentistry, 26(3), 231–239. 15185804

[cre298-bib-0003] Beikler, T. , Schnitzer, S. , Abdeen, G. , Ehmke, B. , Eisenacher, M. , & Flemmig, T. (2006). Sampling strategy for intraoral detection of periodontal pathogens before and following periodontal therapy. Journal of Periodontology, 77(8), 1323–1332. 16881801 10.1902/jop.2006.050204

[cre298-bib-0005] Çalışır, M. , Akpınar, A. , Poyraz, Ö. , Göze, F. , & Çınar, Z. (2015). The histopathological and morphometric investigation of the effects of systemically administered humic acid on alveolar bone loss in ligature‐induced periodontitis in rats. Journal of Periodontal Research, Epub ahead of printing. https://doi.org/10.1111/jre.12329 10.1111/jre.12329 26547279

[cre298-bib-0006] Casas, A. , Herrera, D. , Martín‐Carnes, J. , González, I. , O'Connor, A. , & Sanz, M. (2007). Influence of sampling strategy on microbiologic results before and after periodontal treatment. Journal of Periodontology, 78, 1103–1112. 17539725 10.1902/jop.2007.060232

[cre298-bib-0008] de Molon, R. S. , Mascarenhas, V. I. , de Avila, E. D. , Finoti, L. S. , Toffoli, G. B. , Spolidorio, D. M. , … Cirelli, J. A. (2015). Long‐term evaluation of oral gavage with periodontopathogens or ligature induction of experimental periodontal disease in mice. Clinical Oral Investigations, Epub ahead of printing. https://doi.org/10.1007/s00784‐015‐1607‐0 10.1007/s00784-015-1607-0 26411857

[cre298-bib-0009] Dewhirst, F. E. , Chen, T. , Izard, J. , Paster, B. J. , Tanner, A. C. , Yu, W. H. , … Wade, W. G. (2010). The human oral microbiome. Journal of Bacteriology, 192(19), 5002–5017. 20656903 10.1128/JB.00542-10 PMC2944498

[cre298-bib-0010] Fine, D. H. (2009). Of mice and men: Animal models of human periodontal disease. Journal of Clinical Periodontology, 36(11), 913–914. https://doi.org/10.1111/j.1600‐051X.2009.01456.x 19735469 10.1111/j.1600-051X.2009.01456.x

[cre298-bib-0011] Fontana, C. R. , Kurachi, C. , Mendonça, C. R. , & Bagnato, V. S. (2004). Microbial reduction in periodontal pockets under exposition of a medium power diode laser: An experimental study in rats. Lasers in Surgery and Medicine, 35(4), 263–268. 15493030 10.1002/lsm.20039

[cre298-bib-0012] Graves, D. T. , Fine, D. , Teng, Y. T. , Van Dyke, T. E. , & Hajishengallis, G. (2008). The use of rodent models to investigate host‐bacteria interactions related to periodontal diseases. Journal of Clinical Periodontology, 35(2), 89–105. https://doi.org/10.1111/j.1600‐051X.2007.01172.x 18199146 10.1111/j.1600-051X.2007.01172.x PMC2649707

[cre298-bib-0013] Guentsch, A. , Kramesberger, M. , Sroka, A. , Pfister, W. , Potempa, J. , & Eick, S. (2011). Comparison of gingival crevicular fluid sampling methods in patients with severe chronic periodontitis. Journal of Periodontology, 82(7), 1051–1060. 21235330 10.1902/jop.2011.100565 PMC3129431

[cre298-bib-0014] Guessous, F. , Huynh, C. , N'Guyen, H. , Godeau, G. , Giroud, J. P. , Meyer, J. , … Roch‐Arveiller, M. (1994). An animal model for the assessment of gingival lesions. Journal of Pharmacological and Toxicological Methods, 32(3), 161–167. 7858310 10.1016/1056-8719(94)90070-1

[cre298-bib-0015] Haffajee, A. D. , Cugini, M. A. , Tanner, A. , Pollack, R. P. , Smith, C. , Kent, R. L. Jr. , & Socransky, S. S. (1998). Subgingival microbiota in healthy, well‐maintained elder and periodontitis subjects. Journal of Clinical Periodontology, 25(5), 346–353. 9650869 10.1111/j.1600-051x.1998.tb02454.x

[cre298-bib-0017] Johnson, I. H. (1975). Effects of local irritation and dextran sulfate administration on the periodontium of the rat. Journal of Periodontal Research, 10(6), 332–345. 129558 10.1111/j.1600-0765.1975.tb00042.x

[cre298-bib-0018] Klausen, B. (1991). Microbiological and immunological aspects of experimental periodontal disease in rats: A review article. Journal of Periodontology, (1), 59–73. 2002433 10.1902/jop.1991.62.1.59

[cre298-bib-0021] Loomer, P. M. (2004). Microbiological diagnostic testing in the treatment of periodontal diseases. Periodontology 2000 2000, 34, 49–56. 14717855 10.1046/j.0906-6713.2002.003424.x

[cre298-bib-0023] Persson, G. R. , Weibel, M. , Hirschi, R. , & Katsoulis, J. (2008). Similarities in the subgingival microbiota assessed by a curet sampling method at sites with chronic periodontitis. Journal of Periodontology, 79(12), 2290–2296. 19053919 10.1902/jop.2008.080142

[cre298-bib-0026] Semenoff, S. A. , Semenoff, T. A. , Borges, A. H. , Pedro, F. L. , & Sakai, V. T. (2010). Methodological model of chronic stress associated with ligature‐induced periodontitis in rats: A radiographic study. Brazilian Oral Research, 24(4), 455–459. 21180968 10.1590/s1806-83242010000400014

[cre298-bib-0027] Socransky, S. S. , Haffajee, A. D. , Cugini, M. A. , Smith, C. , Kent Jr., R. L . (1998). Microbial complexes in subgingival plaque. Journal of Clinical Periodontology, 25, 134–144. 9495612 10.1111/j.1600-051x.1998.tb02419.x

[cre298-bib-0029] Socransky, S. S. , Smith, C. , Martin, L. , Paster, B. J. , Dewhirst, F. E. , & Levin, A. E. (1994). "Checkerboard" DNA‐DNA hybridization. BioTechniques, 17(4), 788–792. 7833043

[cre298-bib-0030] Struillou, X. , Boutigny, H. , Soueidan, A. , & Layrolle, P. (2010). Experimental animal models in periodontology: A review. The Open Dentistry Journal, 4, 37–47. 20556202 10.2174/1874210601004010037 PMC2885595

[cre298-bib-0032] Tanner, A. C. R. , & Goodson, J. M. (1986). Sampling of microorganisms associated with periodontal disease. Oral Microbiology and Immunology, 1, 15–20. 3295677 10.1111/j.1399-302x.1986.tb00310.x

[cre298-bib-0033] Teles, F. R. , Haffajee, A. D. , & Socransky, S. S. (2008). The reproducibility of curet sampling of subgingival biofilms. Journal of Periodontology, 79(4), 705–713. 18380565 10.1902/jop.2008.070424

[cre298-bib-0034] Trindade, F. , Oppenheim, F. G. , Helmerhorst, E. J. , Amado, F. , Gomes, P. S. , & Vitorino, R. (2014). Uncovering the molecular networks in periodontitis. Proteomics. Clinical Applications, 8(9–10), 748–761. 24828325 10.1002/prca.201400028 PMC4426160

